# Histopathological Diagnostic Value of the IgG4^+^/IgG^+^ Ratio of Plasmacytic Infiltration for IgG4-Related Diseases: A PRISMA-Compliant Systematic Review and Meta-Analysis

**DOI:** 10.1097/MD.0000000000000579

**Published:** 2015-03-06

**Authors:** Chuiwen Deng, Wenli Li, Si Chen, Wen Zhang, Jing Li, Chaojun Hu, Xiaoting Wen, Fengchun Zhang, Yongzhe Li

**Affiliations:** From the Department of Rheumatology and Clinical Immunology (CD, SC, WZ, JL, CH, XW, FZ, YL), Peking Union Medical College Hospital, Chinese Academy of Medical Sciences and Peking Union Medical College, Key Laboratory of Rheumatology and Clinical Immunology, Ministry of Education; and Department of Rheumatology (WL), China–Japan Friendship Hospital, Beijing, China.

## Abstract

This article aims to perform a meta-analysis to evaluate the diagnostic value of the immunoglobulin G (IgG)4^+^/IgG^+^ ratio of plasmacytic infiltration for IgG4-related diseases.

Four databases—EMBASE, ISI Web of Knowledge, PubMed, and the Cochrane Library—were systematically searched. Approximately 200 participants from several studies were included in this research. STATA 11.2 software (Stata Corporation, College Station, TX) and Meta-DiSc 1.4 (Unit of Clinical Biostatistics, Ramon y Cajal Hospital, Madrid, Spain) were used to perform the meta-analysis.

Nine studies were included in the meta-analysis. The pooled diagnostic odds ratio was 18.94 [95% confidence interval (CI), 2.89–124.30]. The sensitivity was 58.80% (95% CI, 50.90–66.30) and the specificity was 90.20% (95% CI, 81.20–95.80). The positive and negative likelihood ratios were 3.12 (95% CI, 1.07–9.16) and 0.26 (95% CI, 0.09–0.70), respectively. The area under the curve of the summary receiver-operating characteristic was 0.88.

To conclude, the IgG4^+^/IgG^+^ ratio of plasmacytic infiltration is modestly effective in diagnosing IgG-related disease.

## INTRODUCTION

Immunoglobulin G (IgG)4-related disease (IgG4-RD) is a systemic immune-mediated disease characterized by storiform fibrosis, infiltration of IgG4^+^ plasma cells in involved organs, and, in some cases, elevated serum IgG4 levels.^1^ IgG4-RD can affect almost any organ and common sites of involvement are the pancreas, salivary glands, orbit, and lymph nodes. Few studies have focused on the incidence or prevalence of IgG4-RD, but recent epidemiological studies have shown that approximately 6700 to 26,000 patients have developed IgG4-RD over the past 20 years in Japan.^2^

Diagnosis of IgG4-RD mainly depends on 4 criteria: histopathology, imaging, serology, and response to steroid therapy. However, these criteria are insufficient for diagnosing IgG4-RD. Imaging of involved organs cannot efficiently differentiate tumors and IgG4-RD.^3^ Furthermore, elevated serum IgG4 is not exclusively observed in IgG4-RD, and 30% of IgG4-RD patients indeed present normal IgG4 levels.^4,5^ Histopathological evidence has shown higher sensitivity and specificity for steroid therapy, and is widely accepted as the gold standard for the diagnosis of IgG4-RD.^6^

Histopathological findings of IgG4^+^ plasma cells are the most important features of IgG4-RD, especially the IgG4^+^/IgG^+^ ratio. Over the past decade, numerous studies have evaluated the IgG4^+^/IgG^+^ ratio for its ability to accurately diagnose IgG4-RD. However, inconsistent conclusions relating to the diagnostic performance of IgG4^+^/IgG^+^ ratio antibodies have been drawn.^7–15^ The aim of this study was to systematically review the literature to determine the diagnostic performance of the IgG4^+^/IgG^+^ ratio in patients with IgG4-RD.

## METHODS

### Literature Search

Studies were identified in the EMBASE, ISI Web of Knowledge, PubMed, and the Cochrane Library databases. To retrieve all relevant publications related to histopathological findings of IgG4^+^ plasma cells, we searched for the follow terms: “IgG4/IgG ratio,” “IgG4^+^/IgG^+^ ratio,” “ratio of IgG4^+^/IgG^+^,” and “IgG4^+^/IgG^+^” combined with IgG4-related diseases. No limits were placed on ethnicity or geographic region, and all documents were updated to September 2014. Additional relevant references cited in searched articles were also selected. All analyses of this systemic review were based on previous published studies, thus no ethical approval and patient consent are required.

### Eligibility Criteria

Studies meeting the following criteria were eligible for inclusion: those that assessed the diagnostic accuracy of histopathological findings of IgG4^+^ plasma cells with an IgG4^+^/IgG^+^ ratio ≥0.40 and IgG4^+^/high power fields (HPFs) >10 for IgG4-RD; sufficient data reported to construct 2-by-2 tables; no criteria for published language; and studies based on animals or cell cultures and case reports; conference abstracts without subsequent publication in full text were excluded. In the case of overlapping studies, only the study with the largest sample size was included in our analysis.

### Data Extraction

Data were extracted from all selected studies by 2 independent investigators. Interresearcher disagreements were resolved by consensus or by a third investigator. The following data were collected from each selected study: first author's name; publication year; country in which the study was performed; study design; and study results. Study quality was assessed using the Quality Assessment of Diagnostic Accuracy Studies (QUADAS) tool. Authors of the identified studies were contacted via e-mail if further study details were needed.

### Statistical Analysis

Statistical analysis was performed using STATA 11.2 software (Stata Corporation, College Station, TX, USA) and Meta-DiSc 1.4 (Unit of Clinical Biostatistics, Ramon y Cajal Hospital, Madrid, Spain). Heterogeneity between studies was evaluated by Cochrane *Q*-statistic as well as *I*^2^-statistic. A *P* value >0.10 in *Q*-statistic indicated lack of heterogeneity among studies. *I*^2^ <25% was considered low heterogeneity, 25% to 50% moderate, and >50% a high degree of inconsistency. Finally, the overall or pooled diagnostic odds ratio (DOR), sensitivity, specificity, positive likelihood ratio (LR+) and negative likelihood ratio (LR–), and their 95% CIs were obtained by a random-effects or a fixed-effects model in the presence (*P* ≤ 0.10 or *I*^2^ > 50%) or absence (*P* > 0.10 and *I*^2^ ≤ 50%) of heterogeneity, respectively. The area under the summary receiver-operating characteristic (SROC) curves represented the overall performance of the detection method. A *P* value <0.05 (2 sided) was considered significant. Evaluation of threshold effect and publication bias was also undertaken.

## RESULTS

### Literature Search

**FIGURE 1:**
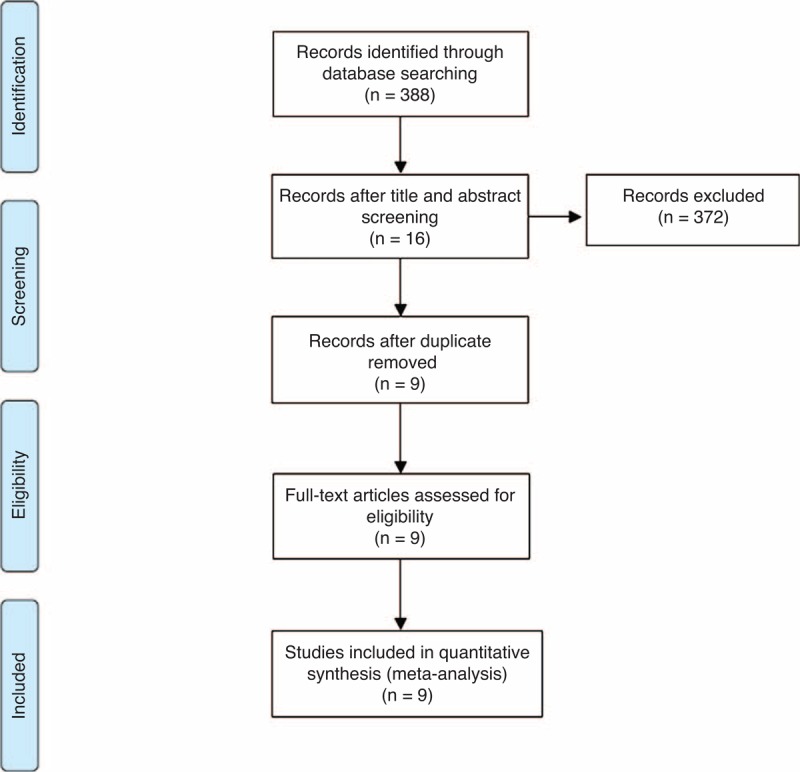
Flow chart of studies included in the meta-analysis.

Electronic and manual searches yielded a total of 388 potentially eligible articles. A flow chart of screening articles for meta-analysis is illustrated in Figure 1. Three hundred seventy-two articles were excluded by screening the titles and abstracts. A further 7 duplicated articles were excluded. A total of 9 eligible studies were included in the meta-analysis.^7–15^

### Study Characteristics

**FIGURE 2:**
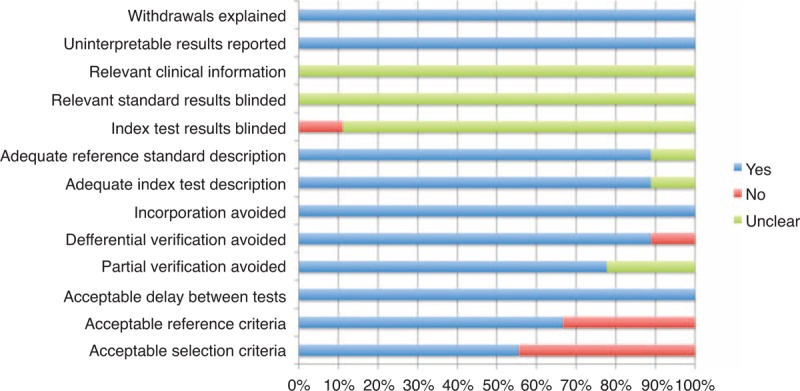
Quality assessment of included studies based on the Quality Assessment of Diagnostic Accuracy Studies tool.

The characteristics of the 9 studies are summarized in Table 1 . A total of 170 IgG4-RD patients and 90 controls were involved in these studies. With regard to the geographic location of the studies, 2 were carried out in the United States,^7,8^ 6 in Japan,^9–11,13–15^ and 1 in Korea.^12^ Assessment using QUADAS indicated that the studies were of medium quality, with positive results for at least 6/14 items (Figure 2).

**TABLE 1:**
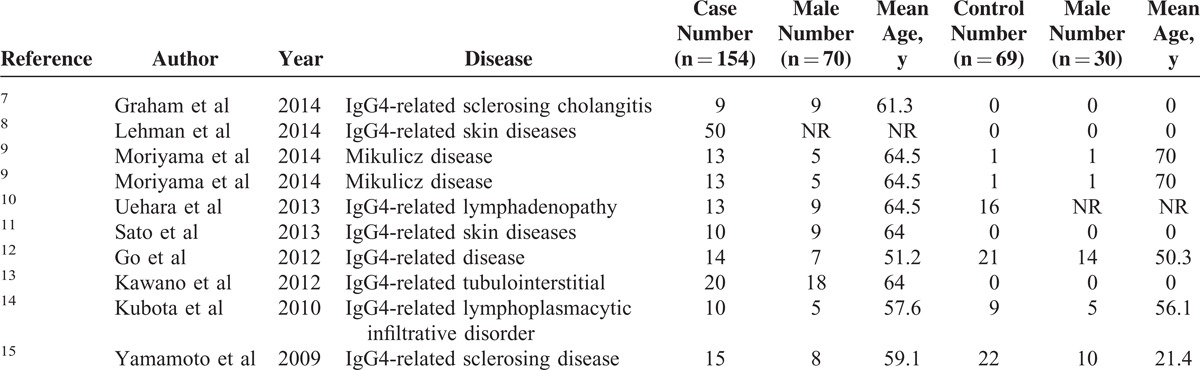
Characteristics of Studies Included in the Meta-Analysis of Diagnostic Performance of IgG4^+^/IgG^+^ Ratio in IgG4-RD

**TABLE 1 (Continued):**
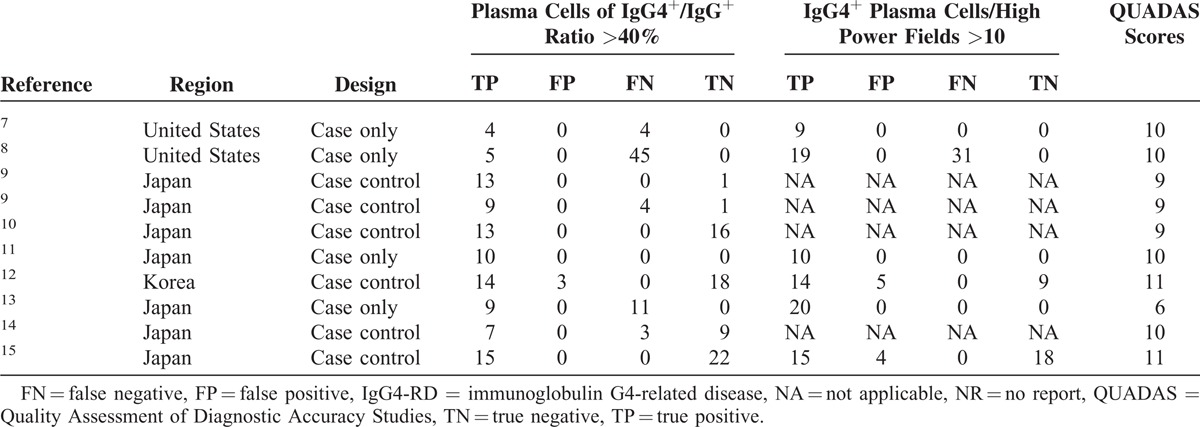
Characteristics of Studies Included in the Meta-Analysis of Diagnostic Performance of IgG4^+^/IgG^+^ Ratio in IgG4-RD

### Meta-Analysis of IgG4^+^/IgG^+^ Ratio of Plasmacytic Infiltration

**FIGURE 3:**
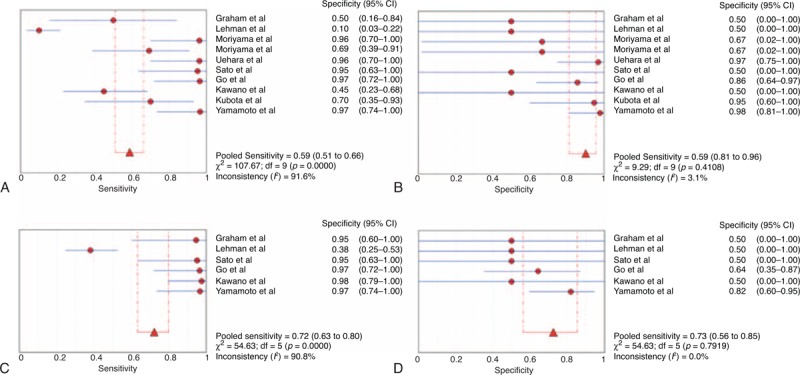
Forests plot of the accuracy of IgG4^+^/IgG^+^ ratio and IgG4^+^/HPF for the diagnosis of IgG4-RD. (A) Sensitivity forest plot of the IgG4^+^/IgG^+^ ratio. (B) Specificity forest plot of the IgG4^+^/IgG^+^ ratio. (C) Sensitivity forest plot of the IgG4^+^/HPF. (D) Specificity forest plot of the IgG4^+^/HPF. HPF = high power field, IgG4-RD = immunoglobulin G4-related disease.

**FIGURE 4:**
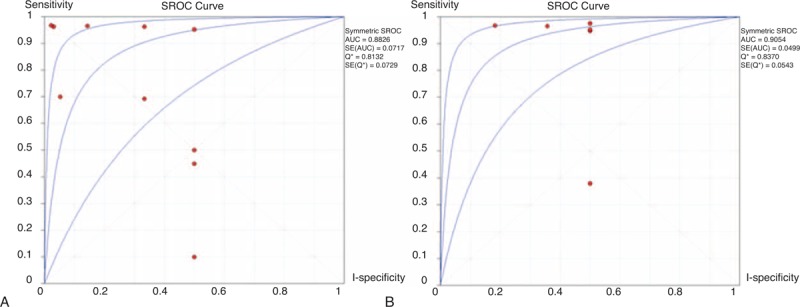
SROCs of IgG4^+^/IgG^+^ ratio and IgG4^+^/HPF for IgG4-RD diagnosis. (A) SROC for IgG4^+^/IgG^+^ ratio. (B) SROC for IgG4^+^/HPF. HPF = high power field, IgG4-RD = immunoglobulin G4-related disease, SROC = summary receiver-operating characteristic.

The sensitivity of the IgG4^+^/IgG^+^ ratio of plasmacytic infiltration ranged from 10.00% to 96.80%, and the reported specificity ranged from 50.00% to 97.80%. The pooled DOR was 18.94 (95% CI, 2.89–124.30; *Q* = 21.34, *P* = 0.01; *I*^2^ = 57.80%). The sensitivity was 58.80% (95% CI, 50.90–66.30; *Q* = 107.67, *P* < 0.01; *I*^2^ = 91.60%) and the specificity was 90.20% (95% CI, 81.20–95.80; *Q* = 9.29, *P* = 0.41; *I*^2^ = 3.10%). The LR+ and LR− were 3.12 (95% CI, 1.07–9.16; *Q* = 24.06, *P* = 0.04; *I*^2^ = 62.60%) and 0.26 (95% CI, 0.09–0.70; *Q* = 21.87, *P* = 0.01; *I*^2^ = 58.90%), respectively. The area under the curve (AUC) of the SROC was 0.88. The forest plots and SROC are shown in Figure 3A and B, and Figure 4A, respectively.

### Meta-Analysis of IgG4^+^ Plasma Cells/HPF

In order to compare the IgG4^+^/IgG^+^ ratio of plasmacytic infiltration with IgG4^+^ plasma cells/HPF, we performed meta-analysis of IgG4^+^ plasma cells/HPF based on the articles included in this research. The sensitivity of IgG4^+^ plasma cells/HPF ranged from 38.00% to 97.60%, and reported specificity ranged from 50.00% to 81.80%. The pooled DOR was 26.16 (95% CI, 5.66–120.95; *Q* = 4.83, *P* = 0.44; *I*^2^ = 0%). The sensitivity was 72.20% (95% CI, 63.30–80.00; *Q* = 54.63, *P* = 0.00; *I*^2^ = 90.80%) and the specificity was 72.50% (95% CI, 56.10–85.40; *Q* = 2.40, *P* = 0.79; *I*^2^ = 0%). The LR+ and LR− were 3.08 (95% CI, 1.91–4.96; *Q* = 4.15, *P* = 0.53; *I*^2^ = 0%) and 0.09 (95% CI, 0.03–0.31; *Q* = 7.39, *P* = 0.19; *I*^2^ = 32.30%), respectively. The AUC of the SROC was 0.91. The forest plots and SROC are shown in Figure 3C and D, and Figure 4B, respectively.

### Exploration of Threshold Effect

A Spearman rank correlation was performed to confirm the threshold effect; indication of no threshold effect was found [Spearman correlation coefficient = −0.77, *P* = 0.009; the slope (b) of the regression equation did differ from zero (*P* = 0.412)].

### Publication Bias

**FIGURE 5:**
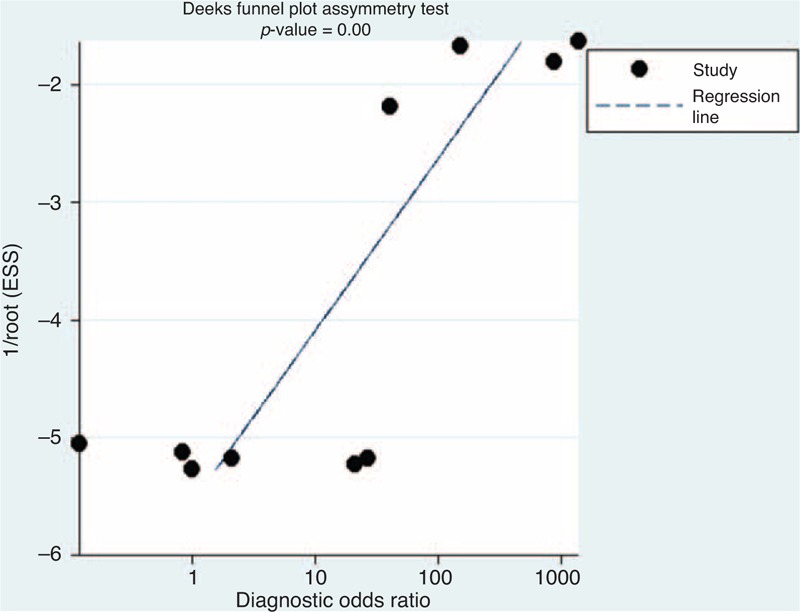
Funnel plot of included studies.

The presence of a statistically significant slope coefficient (*P* < 0.05) was considered to indicate possible bias. We conducted funnel plots that represented a somewhat symmetric curve (Figure 5). The *P* value of the slope coefficient was calculated to be 0.00, indicating that publication bias was observed in the included studies.

## DISCUSSION

The diagnosis of IgG4-RD mainly relies on a triad of clinical characteristics: results of serum IgG4 tests, imaging features, and histological assessment. However, the common clinical manifestations are not specific to IgG4-RD. Furthermore, the performance of serum IgG4 is not satisfactory, having moderate specificity and sensitivity.^4,5^ Histopathological findings were considered the gold standard of IgG4^+^ plasma cells, especially the IgG4^+^/IgG^+^ ratio and IgG4^+^/HPF. To date, no systematic evaluation of the IgG4^+^/IgG^+^ ratio has been performed. To our knowledge, this study is the first meta-analysis to provide precise and controlled data on the diagnostic performance of the IgG4^+^/IgG^+^ ratio in IgG4-RD.

The IgG4^+^/IgG^+^ ratio was found to be a potential diagnostic biomarker of IgG4-RD, and its diagnostic performance needs to be confirmed. There were 9 eligible studies of medium quality (QUADAS scores >6, Figure 2) included in this meta-analysis (Table 1). The pooled specificity was high (90.20%), in comparison with a relatively low sensitivity (58.80%), which revealed that the diagnostic performance of the IgG4^+^/IgG^+^ ratio could identify controls better than IgG4-RD. The SROC curve implies that the IgG4^+^/IgG^+^ ratio showed moderate diagnostic performance for IgG4-RD. The pattern of the data points in the SROC curve did not suggest a “shoulder-arm” shape, and the AUC of SROC was 0.88. Taken together, these results indicate that the IgG4^+^/IgG^+^ ratio had a modest level of overall diagnostic accuracy for IgG4-RD. Considering that IgG4^+^/HPF was also regarded as an important histological feature of IgG4-RD diagnosis, we also performed a meta-analysis of IgG4^+^/HPF. Compared with the IgG4^+^/IgG^+^ ratio, the pooled specificity of IgG4^+^/HPF was lower (72.50%) but the sensitivity was higher (72.20%). The AUC of SROC was 0.91. Primary comparison revealed that the diagnostic performance of IgG4^+^/HPF was better than that of the IgG4^+^/IgG^+^ ratio.

Heterogeneity has been found in the pooled DOR of the IgG4^+^/IgG^+^ ratio (18.94, *P* = 0.01; *I*^2^ = 57.80%). In order to explore the heterogeneity found across studies, publication bias and Spearman rank correlation were performed. No statistical difference was found using Spearman rank correlation, which means that there was no threshold effect among these studies. However, publication bias was found in this meta-analysis, which could introduce heterogeneity.

Some limitations in this meta-analysis need to be noted. The cutoff value of the IgG4^+^/IgG^+^ ratio remains somewhat controversial, and exploring its clinical significance might help further our understanding of the IgG4^+^/IgG^+^ ratio; however, relatively few studies have focused on this aspect. Second, meta-analysis of IgG4^+^ plasma cells/HPF only considered the studies that we included for the IgG4^+^/IgG^+^ ratio, and thus, requires further research.

In conclusion, the IgG4^+^/IgG^+^ ratio is a modestly effective diagnostic biomarker for IgG4-RD, with a low sensitivity but a high specificity.
